# Detection of dairy products from multiple taxa in Late Neolithic pottery from Poland: an integrated biomolecular approach

**DOI:** 10.1098/rsos.230124

**Published:** 2023-03-15

**Authors:** Miranda Evans, Jasmine Lundy, Alexandre Lucquin, Richard Hagan, Łukasz Kowalski, Jarosław Wilczyńki, Penny Bickle, Kamil Adamczak, Oliver E. Craig, Harry K. Robson, Jessica Hendy

**Affiliations:** ^1^ Department of Archaeology, University of York, Heslington YO10 5DD, UK; ^2^ Department of Archaeology, The University of Cambridge, Cambridge CB2 3DZ, UK; ^3^ Institute of Archaeology, Centre for Applied Archaeology, Nicolaus Copernicus University in Toruń, Szosa Bydgoska 44/48, 87-100 Toruń, Poland; ^4^ Department of Vertebrate Zoology, Institute of Systematics and Evolution of Animals, Polish Academy of Sciences, Sławkowska 17, 31-016 Kraków, Poland

**Keywords:** palaeoproteomics, lipids, organic residue analysis, Funnel Beaker culture, ceramics

## Abstract

The detection of dairy processing is pivotal to our understanding of ancient subsistence strategies. This culinary process is linked to key arguments surrounding the evolution of lactase persistence in prehistory. Despite extensive evidence indicating the presence of dairy products in ceramics in the European Neolithic, questions remain about the nature and extent of milk (and lactose) processing and consumption. In order to investigate past patterns of dairy processing, here we analyse ancient proteins identified from Late Neolithic Funnel Beaker ceramics, scrutinizing the principle that curd and whey proteins partition during the production of dairy foods from milk. Our results indicate the presence of casein-rich dairy products in these vessels suggesting the creation of curd-enriched products from raw milk. Moreover, this analysis reveals the use of multiple species for their dairy products in the Late Neolithic, adding to a growing body of evidence for the period. Alongside palaeoproteomic analysis, we applied well-established lipid residue analysis. Differential interpretations between these two approaches show that palaeoproteomics is especially useful where the effects from isotope mixing may underestimate the frequency of dairy products in archaeological ceramics, highlighting the potential utility of a multi-stranded approach to understand life histories of vessel use.

## 1. Introduction

Cheese making is a unique and important culinary invention, enabling milk to be preserved, stored and transported while offering considerable nutritional value, containing protein, sugars and fats. The detection of cheese making in the past has substantial implications for understanding choices surrounding the use of primary and secondary products in daily life, for personal preferences and cultural identities [1–3]. Moreover, identifying the species used for dairy production informs us about environmental niches and pastoral strategies, e.g. [4,5]. The link between dairy consumption, processing and lactase persistence (LP) evolution has also received extensive attention and debate, and it has been proposed that cheese making may have been used as a strategy for reducing the lactose content of milk products, thus rendering foods rich in protein and fat digestible to lactase non-persistence individuals [6]. While the use of dairy products has been extensively shown throughout Neolithic Europe through faunal mortality patterns, lipid residue analysis and ancient dental calculus [7–13], recent research suggests that the presence of dairying does not necessarily correspond with LP allele frequency trajectories [9], and other research has demonstrated the consumption of dairy foods in individuals likely to be non-lactase persistent, e.g. [13,14]. Therefore, questions remain about the extent and nature of dairy processing in relation to lactose content.

### 1.1. Dairying in the Funnel Beaker culture

A growing body of archaeological evidence demonstrates that milk was consumed from the beginning of the Neolithic in Central Europe. The presence of dairy lipids in pottery from Hungary and Poland dating to the sixth millennium BCE supports the idea that dairying was part of the cultural package travelling with early farmers from the Near East to Europe. There is little evidence, however, that milk formed a significant component of the diet during the earliest Neolithic, but was rather consumed occasionally as a product of a broad mixed economy [6,12,15–17]. Many studies have suggested that this pattern of milk consumption changed profoundly during the mid-fourth millennium BCE, when a focus on secondary animal products becomes visible in mortality profiles and sex ratios of herds [12]. There are strong archaeological indications to suggest that the later generations of the Funnel Beaker (*Trichterbecherkultur*, hereafter TRB) farmers of north-central Europe saw this shift towards a more diversified exploitation of domestic cattle and ovicaprinae, which is often summed up as a Secondary Products Revolution [18–21].

In Poland, the traditional standpoint has been that the subsistence economy of the TRB was focused on domestic livestock exploitation for primary (meat, hide and bone) and secondary products (milk, wool and animal traction), and only occasionally did their economic repertoire also include hunting and gathering [19]. Although the presence of ceramic strainers that could be used to separate curds and whey seem to point to dairying in the TRB, the respective age-at-death profiles have generally proven scarce [22,23]. However, the positive indication for intense milk production in the region of modern Poland can be identified through the sex ratios of cattle herds, showing that in the later stages of the Neolithic, the number of cows is twice the number of bulls compared with the herding structures at the beginning of the Neolithic [24]. Perhaps the most direct evidence for the use of dairy comes from the lipid residue analysis of TRB ceramics from Kopydłowo, central Poland [25]. It is clear that a considerable portion of ceramic beakers recovered from the site were the prime containers for milk, and we now know that a small-scale dairy economy was present from the onset of the TRB in some regions, such as the western Baltic and Southern Scandinavia [7,10,26,27].

### 1.2. Organic residue analysis and dairying

The analysis of lipids extracted from ancient pottery has been instrumental in providing direct evidence for dairying in the archaeological record. Previous research has revealed the association between dairy fats and ceramic vessels in Southern Europe and the Near East [28,29], sub-Saharan Africa [30] and Northern Europe [6]. Salque *et al*. [6] argue that the presence of dairy lipid residues present in ceramic sieves demonstrates their specialized use in cheese production. Differences in fatty acid isotope values have been reported between fermented and unfermented dairy products [31], although these are more likely explained by variation in modern farming practices, including maize silage in animal feed [32]. Currently there are no reliable molecular or isotopic criteria to distinguish lipid residues derived from raw milk from those derived from fermented dairy products such as cheese, yoghurt or kefir.

The analysis of milk proteins has also emerged as a powerful tool for understanding ancient dairying and dairy consumption, with ceramic vessels and their residues, and ancient dental calculus often targeted. For example in ceramics, immunological methods revealed dairy proteins from the Early Iron Age Outer Hebrides [16], and more recently liquid chromatography tandem mass spectrometry (LC-MS/MS)-based proteomics was applied to ceramic limescale deposits from the sites of Çatalhöyük West [33], Bah̄rija [34] and two sites in the Campania Region [35]. With results from Çatalhöyük West revealing that mixing occurred between sheep and goat dairy products and a range of plant foods [33]. Specifically, this also revealed cow dairy and milk whey processing occurred in specific vessels, potentially indicating specialized food preparation processes [33]. Proteomic analyses of ancient dental calculus have frequently produced taxonomically specific information on dairy consumption, as opposed to processing, with the whey protein beta-lactoglobulin (BLG) being commonly preserved [36]. This approach has now been applied to a range of contexts, revealing the consumption of dairy foods in victims of the Great Irish Famine [37], Neolithic Britain [13], Neolithic-Meroitic Eastern Africa [38] and the East Eurasian Steppe [14].

### 1.3. Milk processing and the milk proteome

**Figure 1.**
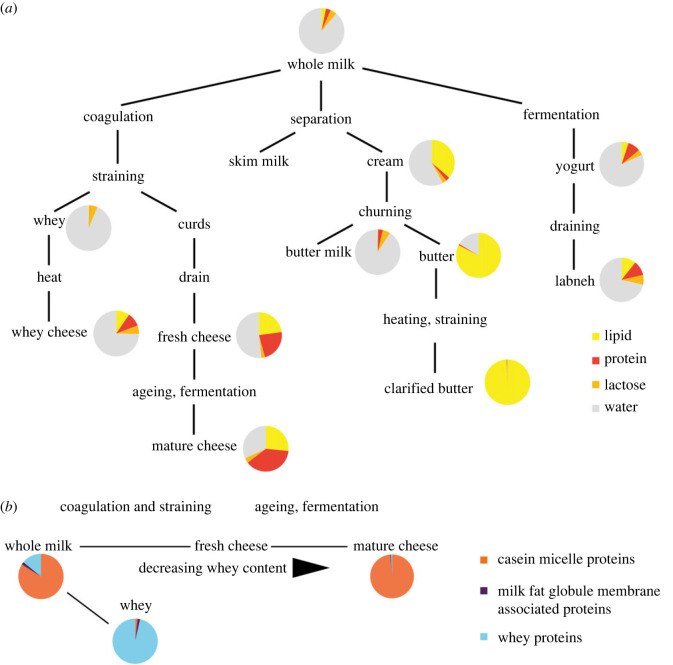
(*a*) Simplified model of a range of dairy processing methods showing the change in the proportion of lipid, protein, lactose and water content (after figure 39 in [39] using nutritional data (electronic supplementary material, table S1)). (*b*) Simplified model of change in the proportion of different dairy protein fractions (casein micelle proteins, milk fat globule membrane-associated proteins and whey proteins) during the process of cheese making (data from [40]).

The processing of milk into dairy foods results in characteristic changes to protein abundance and content (figure 1). For example, in rennet-based cheese making, casein micelles (spherical aggregates of four different casein proteins) coagulate when kappa-casein in the micelle reacts with rennet (composed primarily of chymosin, itself a protein). This results in the separation of these coagulated casein proteins from water-soluble proteins found in the whey, such as BLG, alpha-lactalbumin and lactotransferrin, which are left in the whey solution together with much of the milk sugar (lactose) and fats. With the growing applicability of palaeoproteomics to studies of ancient dairying, there is an opportunity to explore to what extent these biochemical processes inherent in dairy processing can be identified in archaeological substrates. This would enable a better understanding of the nature and extent of ancient dairying processes and culinary practices associated with this key food. Moreover, identification of processed dairy products also informs us more precisely about the degree of lactose consumption of ancient populations and its relationship to LP selection.

Some initial attempts exploring how specific milk proteins may be related to dairy processing in archaeological contexts have been undertaken, but the results remain inconclusive. Warinner *et al*. [36] note that the presence of the whey protein, BLG, may act as a proxy for lactose consumption, given that BLG partitions with lactose during cheese coagulation. Charlton *et al*. [13], Jeong *et al*. [14] and Bleasdale *et al*. [38] expanded this approach to British, Central Asian and East African contexts, respectively, where BLG was detected in individuals who were, or were likely to be lactase non-persistent, suggesting the consumption of lactose by individuals with no evidence for LP alleles. Hendy *et al*. [33], analysing ceramic vessels, observed the presence of whey proteins in a Neolithic ceramic jar which may indicate the presence of this liquid fraction, as opposed to raw milk. Examining well-preserved whole dairy products from Early Bronze Age China, Yang *et al*. [41] studied the cleavage patterns of kappa-casein, a protein integral in casein micelle aggregation. Detecting a lack of bond cleavage due to rennet, and the presence of *Lactobacillus kefiranofaciens* protein, they suggest that these products were created by a kefir-based production. Although this body of literature has revealed that palaeoproteomics is a useful approach for detecting ancient dairying, it has also revealed that there are preservation biases that may impact our ability to accurately interpret dairy processing. For example, Hendy *et al*. [42] observed a high abundance of the milk whey protein BLG compared with other dietary proteins in ancient dental calculus, despite the fact that casein proteins make up 80–85% of milk protein content [40]. When casein proteins are detected, it is often in well-preserved contexts, such as the arid conditions of Xinjiang, China [41,43], and the cold conditions of Mongolia [5].

In this study, we examined archaeological and modern replica ceramics to explore the degree to which the presence and abundance of milk proteins indicate the production of processed dairy curds. We undertake proteomic analysis of four TRB vessels from the site of Sławęcinek in central Poland [44] and four modern replica analogues of cheese bowls and strainers, where curdled milk products have been created and strained, respectively. In the vessels from Sławęcinek, we also compare the palaeoproteomic results with the analysis of extracted lipids.

## 2. Methods

**Figure 2.**
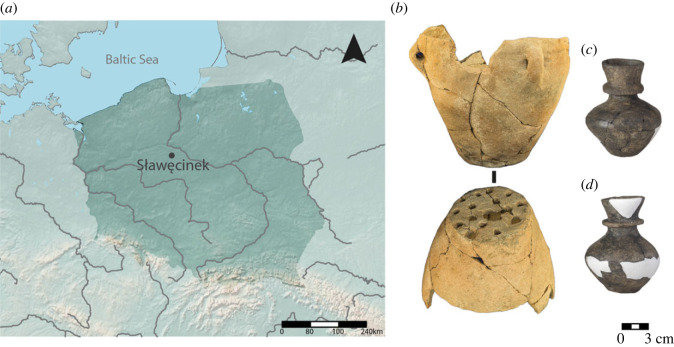
(*a*) Map showing the location of Sławęcinek. (*b*) Ceramic strainer SLA_10 (adapted from [47]). (*c*) Collared flask SLA_1. (*d*) Collared flask SLA_2.

The site of Sławęcinek is located near Inowrocław in central Poland (figure 2) and was discovered during rescue excavations undertaken in 2016. Late Neolithic strata (*ca* 3650–3100 cal BCE) were uncovered, revealing a TRB settlement with four houses, water wells, burials and evidence for both domestic and ritual events at the site [44]. Accelerator mass spectrometry (AMS) radiocarbon dating was undertaken on bone material from the pits, returning the following ages: (i) SLA_3 and 4: 4680 ± 35 BP; 3517–3375 cal BCE at 68.2%; (ii) SLA_8: 4700 ± 30 BP; 3522–3378 cal BCE at 68.2%; and (iii) SLA_10: 4660 ± 40 BP; 3511–3371 cal BCE at 68.2%; all dates presented in this paper were modelled in OxCal v. 4.3.2, using the IntCal20 calibration curve [45,46].

Among the many potsherds unearthed from the site, a small proportion of fragments of collared flasks contained white mineralized deposits adhering to their interior walls (0.2% of the total assemblage). Scanning electron microscopy with energy dispersive X-ray spectroscopy (SEM-EDX) analysis confirmed that the residues are composed of calcium and oxygen (full methodological details on SEM-EDX analysis can be found in the electronic supplementary material, file S1).

Proteomic analysis on the residues adhering to the vessel walls was undertaken on three collared flasks, SLA_3, SLA_4 and SLA_8, and a ceramic strainer, SLA_10 (figure 2*b*; electronic supplementary material, figure S1). Then, lipid residue analysis was undertaken on the same vessels on the ceramic itself, supplemented by a further four collared flasks and two flasks from the assemblage (electronic supplementary material, table S2).

For comparison, proteins were also analysed from residues adhering to four modern vessels where milk had either been warmed and coagulated, or coagulated and strained. The modern comparative assemblage comprises residues from four modern vessels, including two strainers and two bowls (electronic supplementary material, table S2 and figure S2), which were used to make cows' milk cheese using both microbial rennet and acid precipitation for a public outreach archaeology project [48]. In the bowls, milk was warmed to approximately 37°C and coagulated via microbial rennet and acid precipitation, while the strainers were used to strain the curds from the whey, with the coagulation taking place in a separate vessel. Repeated use of these modern replica vessels left visible white residues on their interior surfaces (electronic supplementary material, figure S2), which were visually similar to those on the archaeological samples (electronic supplementary material, figure S1).

### 2.1. Protein extraction and identification

Proteins were extracted separately from the archaeological and modern replica vessels and analysed by LC-MS/MS. Four archaeological vessels were sampled for proteomic analysis, including three collared flasks (SLA_3, SLA_4 and SLA_8) and a strainer (SLA_10). The extraction of all ancient and modern samples was undertaken using a version of the filter-aided sample preparation protocol modified for ancient samples [18] followed by desalting using C18 100 µl pipette tips. Ancient samples were analysed in a laboratory dedicated to ancient samples. Full details of the methodology used for protein extraction, identification and data analysis can be found in the electronic supplementary material, file S1. All samples were analysed by LC-MS/MS at the Centre of Excellence in Mass Spectrometry at the University of York on an Orbitrap Fusion.

To interrogate the data, two data analysis approaches were performed. First, data from the ancient samples were searched using Mascot against all of Swiss-Prot (release: 2020_02) to assess a wide range of potential dietary and non-dietary proteins present (electronic supplementary material, table S3). Then, all ancient and modern samples were searched using MaxQuant v. 1.6.17.0 against a database comprising all annotated dairy protein sequences from any taxa (electronic supplementary material, file S2) and a database of common contaminants (electronic supplementary material, file S3), given the fact that the only putative dietary proteins identified were milk proteins. In MaxQuant, peptides were searched allowing for semi-tryptic cleavage, minimum length of seven, with both a protein and peptide target false discovery rate of 1%. This MaxQuant data forms the basis of the dairy interpretations presented in the study (electronic supplementary material, tables S4 and S5).

Peptides from putative dietary sources were then BLAST searched (NCBI protein–protein BLAST) to establish the lowest common taxonomic ancestor of each identified peptide. Many of the dairy peptides matched both to a milk protein reference and a *Jeotgalicoccus* sequence (a facultatively anaerobic, and halotolerant group, first reported in fermented seafood). Wilkin *et al*. [49] recently noted that the *Jeotgalicoccus* sequence WP_188349304.1 is likely to have high levels of milk contamination from laboratory analysis; as such, *Jeotgalicoccus* was not considered a valid result in taxonomic attribution. While deamidation patterns have been used to investigate the preservation quality of ancient dietary proteins [50], recently the high variability of deamidation patterns in dairy proteins has been demonstrated, rendering their application to samples with low peptide recovery challenging [51]. As such, although deamidation of asparagine and glutamine were detected in this data analysis, this line of enquiry was not used.

### 2.2. Lipid extraction and identification

Absorbed lipids were extracted using an acidified methanol extraction (AE) and characterized using gas chromatography-mass spectrometry (GC-MS) techniques. Full details of the methodology used for lipid extraction, identification and data analysis can be found in the electronic supplementary material, file S1. First, an AE was applied [52,53]. To further understand the origin of these lipids, the extracts were analysed by gas chromatography combustion isotope ratio mass spectrometry (GC-C-IRMS) to determine the stable carbon isotope values of the major fatty acids (C_16:0_ and C_18:0_). This approach has shown to be useful for distinguishing ruminant adipose (i.e. carcass fats), ruminant dairy fats, non-ruminant adipose, as well as marine and freshwater resources based on comparison of the *δ*^13^C values with modern reference values [53–56]. The equipment and instrument parameters are as previously described [57] and are outlined in the electronic supplementary material, file S1.

## 3. Results and discussion

### 3.1. Vessel contents at Sławęcinek

Proteins were recovered from all four TRB vessels, with the majority of protein groups matched to common laboratory and handling contaminants, such as human keratin and other skin-associated proteins which have been previously observed in proteomic analyses of ceramic residues [33,34] (electronic supplementary material, table S3). Proteins detected in extraction blanks used to monitor laboratory contamination included only keratin proteins, trypsin and streptavidin, a protein commonly used in molecular biology laboratories. A positive control of porcine gelatine yielded a high abundance of collagen peptides, indicating the efficacy of the extraction protocol.

**Figure 3.**
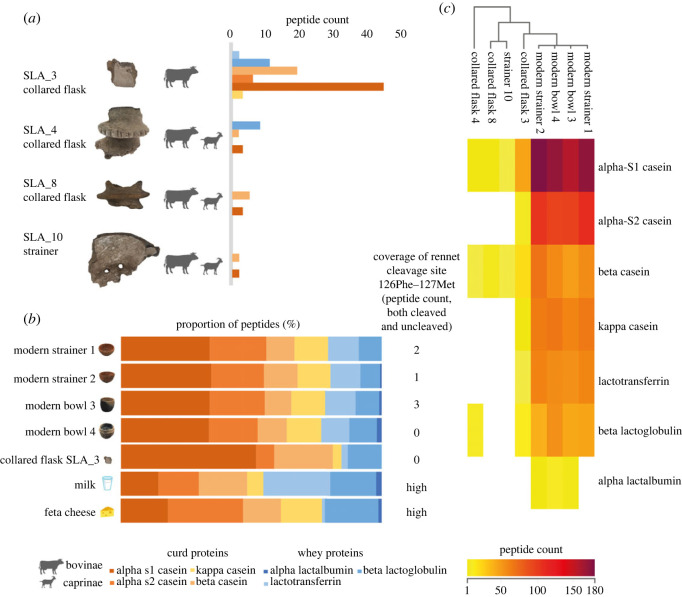
(*a*) Dairy proteins detected in the four TRB vessels from Sławęcinek, indicating the presence of a range of Bovinae and Caprinae milk proteins. (*b*) Proportion of peptides by protein type in the modern cheese-making strainers and pots, ancient sample SLA_3, and modern milk and feta cheese (data from [41]). (*c*) Cluster analysis of the proportion of peptides by protein type present in the modern cheese-making vessels and ancient samples from Sławęcinek (Pearson correlation, Perseus v. 1.6.14.0 [58]), noting the similarity between the two modern strainers and two bowls analysed in this study.

Putative dietary proteins identified in the assemblage were exclusively dairy (figure 3*a*). No meat, plant or yeast proteins were identified.

The proteomic results reveal dairy taxonomy specific to the subfamily level, with evidence of both Caprinae (probably sheep and/or goat) and Bovinae (probably cow) milk. Collared flask SLA_3 bears evidence for Bovinae. However, samples SLA_4, SLA_8 (collared flasks) and SLA_10 (ceramic strainer) all bear evidence specific to the Bovinae subfamily as well as separate evidence for Caprinae (electronic supplementary material, figures S3–S6). This demonstrates that, at least at Sławęcinek, a mixed dairy species economy was being practised during the TRB.

This taxonomic evidence generated by proteomic analysis is congruent with the faunal assemblage recovered from the site, revealing a diversified dairy strategy which used both Bovinae and Caprinae. The number of identified specimens (NISP) and minimum number of individuals (MNI) established for the site strongly support this interpretation. Cattle (*Bos taurus*; NISP = 1404/64.4%, MNI = 33/45.2%), followed by sheep/goat (*Ovis aries/Capra hircus*; NISP = 302/14.1%, MNI = 12/16.4%) and pig (*Sus scrofa f. domestica*; NISP = 283/13.2%, MNI = 19/26%) predominated the assemblage [44].

Further support for milk production at Sławęcinek comes from mandibular mortality profiles of 17 cattle, which shift towards older animals, showing that four of them were immature (*ca* six months of age), five individuals were subadults (*ca* 24 months), and the remaining eight mandibles belonged to adult individuals (greater than 2.5 years). A mixed dairy species strategy at the site is further evidenced by the age-at-death rates for ovicaprinae, with most individuals culled when older; contrary to the exploitation pattern observed for pigs, which points to a focus on a meat-oriented strategy. Overall, the domestic cattle and ovicaprinae management at Sławęcinek signals the intensification in milk production, and it is interesting to note that the nearby TRB site of Kopydłowo produced similar evidence for the incorporation of dairy foods reduced in lactose into the subsistence economy of the region in the mid-fourth millennium BCE [25].

### 3.2. Indicators of dairy processing

A range of dairy proteins was identified in the collared flasks and ceramic strainer analysed in this study, including peptides matching to alpha-S1 and alpha-S2 casein, beta-casein, kappa-casein, BLG and lactotransferrin. Caseins were the most frequently identified class of protein, in particular beta-casein and alpha-S1 casein, both by peptide count and peptide intensities from label-free quantification. Compared with ceramic residues published in Hendy *et al*. [33], the ratio of BLG to casein peptide identification was low.

The dominance of caseins in this assemblage could suggest that the residues formed on these vessels are the result of the presence of casein-rich curd products, rather than milk or whey products. Cheese is composed primarily of curd proteins while the whey proteins and the majority of the lactose remain in the whey portion when the curds coagulate (figure 1). Identification of dietary proteins was especially abundant in collared flask sample SLA_3 compared with the other ancient samples, and here the relative proportion of abundant curd proteins present in sample SLA_3 resembles that seen in experimental soft-curd cheese-making vessels (figure 3*b,c*). We note the contrast in the ancient milk proteome detected in this study compared with other studies identifying milk proteins in ceramic residues [33] and ancient dental calculus [13,38,42], which are dominated by the presence of BLG, a whey protein. We also note that only four vessels were examined, and there is variation in the number of milk peptides detected between vessels. This variability in protein identification has been noted elsewhere [13] and may be due to differences in preservation, pot use, frequency of use or depositional environment.

To further explore whether the proportion of caseins and whey proteins might reflect dairy processing in the vessels, we analysed a set of replica modern vessels used in cheese making. Analysis of the extracted proteins demonstrated that the profiles were not distinctive between the modern bowls and strainers, with the proportion of curd (casein) peptides to whey peptides being similar in the strainers and bowls (figure 3*b*). In contrast with our hypothesis, this indicates that, at least in the modern vessels examined here, there is no discernible difference in the proteins detected (using both peptide count and label-free quantification as metrics for abundance) in vessels used for straining curds versus those where milk has been warmed and coagulated. This could be owing to the mechanisms of protein entrapment in these residues, with further controlled work needed to explore the preservation of milk proteins from a diversity of dairy products in ceramics and ceramic residues. There was, however, a notable difference between the dairy peptide proportion identified in the experimental pottery compared with that of raw milk (data from [41]). The heightened curd protein content of all experimental cheese-making vessels closely resembles that of cheese, differing markedly from the lower curd peptide proportion observed in raw milk (figure 3*b*,*c*), indicating that the identification of particular proteins and peptides in visible residues may be associated with their preservation in milk scale, rather than reflecting the method of processing. Moreover, sample SLA_3, which bears the most abundant peptide evidence of the archaeological samples, similarly resembles the high proportion of curd peptides to whey peptides observed in the modern cheese-making vessels, and in fresh cheese.

Rennet (a set of enzymes derived from ruminant stomachs, primarily the protein chymosin) is commonly used in cheese making to coagulate raw milk into curds. Chymosin causes the cleavage of kappa-casein at ^126^Phe–^127^Met, and therefore, the presence of this cleavage may indicate the use of this curdling technique. The presence of the intact peptide spanning this cleavage site was used to infer that cheese curds preserved from Bronze Age Xiaohe were not generated by ruminant rennet [41]. In the Neolithic samples from Sławęcinek, the relevant portion of the kappa-casein sequence was not present, so here we cannot use this marker to indicate the use of rennet.

However, to examine the efficacy of this approach to detect rennet use in vessel residues, we also examined kappa-casein in the modern cheese-making vessels. We note the very low number of instances of peptides covering the region ^126^Phe–^127^Met, even in the modern cheese-making residues (figure 3*b*,*c*), which together with their absence in the archaeological samples of this study suggests that it is unlikely that rennet detection will be possible in poorly preserved ancient pottery residue samples which have experienced further taphonomic degradation.

### 3.3. Lipid residue analysis

To further enrich our understanding of vessel use, we performed lipid residue analysis on the ceramic powder obtained from the same vessels used to identify proteins and six additional vessels that did not have adhering limescale deposits. All samples yielded interpretable quantities of lipids ranging from 31 to 3057 µg g^−1^ (electronic supplementary material, table S6). Through analysis by GC-MS, the majority of samples yielded profiles typical of degraded animal fats, characterized by an approximately equal dominance of palmitic (C_16:0_) and stearic acids (C_18:0_) in addition to relatively low concentrations of unsaturated fatty acids (C_18:1_). Furthermore, the presence of odd-chain branched fatty acids (C_15:0_ and C_17:0_) in all samples analysed is consistent with the presence of ruminant fats (adipose or dairy) [59]. In addition to degraded animal fats, diterpenoids (e.g. abietic acid) and their oxidation by-products were identified in SLA_10, indicating the presence of *Pinaceae* resin, potentially used as an adhesive or as a waterproofing agent for the vessel [60–62]. A distribution of long odd *n-*chain alkanes with a dominance of *n*-alkane C_27_ and C_29_ (*δ*^13^C −30.0‰ and −34.3‰, respectively) was also identified in SLA_10, indicative of the presence of leafy plants mixed with animal fats and pine products (electronic supplementary material, table S6) [63].

The lipid profiles of samples SLA_1 and SLA_7 are instead indicative of plant oil based on a relatively high oleic to stearic acid ratio (C_18:1_/C_18:0_ greater than 2) and palmitic to stearic acid ratio C_16:0_/C_18:0_ greater than 3. However, rapid degradation of plant oils in the burial environment and through the use of the pot can make it difficult to determine their origin [64].

**Figure 4.**
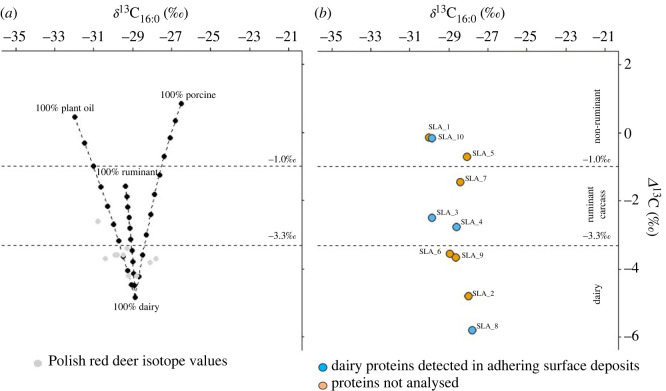
(*a*) Theoretical mixing curves of *δ*^13^C_16:0_ and Δ^13^C values resulting from mixing modern dairy with modern plant oils, porcine fat and ruminant carcass fat, where filled circles are representative of percentage contributions in 10% increments between the mean values. The *δ*^13^C values of modern products used in the mixing model are previously published and reported in [8] or electronic supplementary material, file S4. Grey circles show the *δ*^13^C_16:0_ and Δ^13^C values of modern red deer from Poland [55]. (*b*) Difference in the *δ*^13^C isotope values (Δ^13^C) of the individual mid-chain length fatty acids (C_16:0_ and C_18:0_) obtained from all samples analysed from Sławęcinek. Blue dots indicate samples where dairy proteins were identified in the calcite surface deposits. Δ^13^C values less than −3.3‰ are typically associated with ruminant fats, between −3.3‰ and −1.0‰ are associated with ruminant carcass fats, and above −1.0‰ are associated with non-ruminant fats based on modern reference values [55].

To further understand the origin of the extracted lipids, carbon stable isotope (*δ*^13^C) values of palmitic (C_16:0_) and stearic (C_18:0_) acid were measured and compared with modern reference values (figure 4*b*). Fatty acid stable isotope values from different tissues reflect differences in their biosynthesis. This approach has been particularly useful for identifying ruminant dairy, carcass and non-ruminant lipids in archaeological pottery [65]. Here, two samples (SLA_2 and SLA_8) had Δ^13^C values (defined as *δ*^13^C_18:0_ − *δ*^13^C_16:0_) of less than −4.3‰ and can be said to have been used unequivocally to process/store ruminant dairy products (figure 4*b*). Lipids from a further two samples (SLA_6 and SLA_9) meet the criteria used by Copley *et al*. [65] for dairy products (i.e. less than −3.3‰) but also are within the range of carcass fats from wild ruminants such as red deer [55], which were present in the faunal assemblage at Sławęcinek. Three samples (SLA_3, SLA_4 and SLA_7) have Δ^13^C values outside the dairy range and within the range of modern ruminant adipose fats, i.e. Δ^13^C values greater than −3.3‰ and less than −1.0‰, and the remaining three samples (SLA_1, SLA_5 and SLA_10) are within the range of modern non-ruminant fats (e.g. porcine), i.e. Δ^13^C values greater than −1.0‰. Mixing of fatty acids derived from different products further complicates the interpretation and is illustrated in figure 4*a*.

### 3.4. Comparison between proteins and lipids

This study provided a valuable opportunity to compare lipid and protein approaches for identifying vessel contents, albeit only four vessels were available for comparison using both methods. We note that although four vessels had adhering surface deposits containing dairy proteins, only one (SLA_8) had a lipid Δ^13^C value less than −4.3‰ with conclusive evidence of dairy products [55]. SLA_3 and SLA_4 had Δ^13^C values of −2.5‰ and −2.8‰, respectively, indicating ruminant carcass fats, yet both of these vessels had deposits adhered to their surfaces containing dairy proteins, which were particularly abundant in the case of SLA_3. Contrasting with the protein results, the Δ^13^C value of the ceramic strainer (SLA_10, approx. −0.2‰) is not congruent with ruminant dairy fats and is instead within the range of non-ruminant animal fats or plant oils, with additional lipids suggestive of plant resin and oil. Despite this, we note that the number of dairy peptides in this sample is relatively low compared with the other vessels, and that other potential dietary proteins were not detected.

Interestingly, the results from both the lipid and protein analyses from the vessels do not necessarily align, for example vessel SLA_10 yielded markedly different interpretations from the two approaches. From the lipid results alone, three of the four pots would not have been interpreted as containing dairy, yet milk proteins were detected. A likely explanation for the discrepancy is that the lipids absorbed in the pottery are derived from a mixture of different sources, as has been suggested previously [33,66]. To illustrate, mixing lines between the mean isotope values for dairy and other products are shown in figure 4*a* with 10% increments. From this it is clear that Δ^13^C values outside the dairy range are achievable when substantial amounts of dairy fats are mixed with other fats and oils.

Other explanations for the discrepancy in these two approaches could be that non-dairy proteins may have indeed been part of the vessel's use-life, but that entrapment, decay and extraction have impacted the observed protein extractome. Moreover, in this study, protein analysis was performed on the adhering residue of the vessel, while the lipid extractions were performed on the ceramic itself and therefore these differing results could also be indicative of different periods of the vessel's use-life (as has been shown in foodcrusts [67]). For example, the presence of *Pinaceae* resin in the ceramic of SLA_10 could indicate the use of a sealant, while the presence of milk protein in the adhering residue reflects the vessel's contents.

Nevertheless, the discrepancy between protein- and lipid-based approaches has implications for the interpretation of ancient vessel use which extends beyond this study. For example, using Δ^13^C values less than or equal to −3.1‰ as a proxy for milk fats, Evershed *et al*. [9] built up a detailed picture of dairying across prehistoric Europe through the analysis of over 6500 *δ*^13^C values. Although this proxy is undoubtedly heavily influenced by the presence of dairy fats, the actual frequencies obtained through time and space are almost certainly an underestimation of the actual frequency of milk use in prehistoric pottery as, unlike the protein evidence presented here, Δ^13^C values are strongly influenced by mixing of different food products (figure 4*a*). Significantly, in regions where the majority of pots have Δ^13^C values greater than −3.1‰, such as Northern Greece, seemingly an outlier in the context of Neolithic Europe, the data might reflect differences in culinary practice, such as a greater propensity for mixing of food products in pottery, rather than fundamental differences in the economy. This does not diminish the utility of Δ^13^C values for inferring dairying, although in some cases, as here, wild ruminant carcass fats must be considered as an alternative source [55], and it is not disputed here that this practice was widespread across Neolithic Europe, only that it is difficult to measure dairying intensity without considering the issue of mixing [8].

Both lipid- and protein-based approaches have methodological strengths. One methodological advantage of palaeoproteomics is that it is possible to identify a degree of taxonomic specificity, owing to key single amino acid polymorphism in the milk proteins of different taxa. Here, as in other studies [5], this characteristic facilitates the identification of dairy from multiple different species. Second, a potential strength of palaeoproteomics is its ability to detect multiple different proteins present in dairy, a property explored in this study to investigate the possibility that an abundance of curd proteins relative to whey proteins may indicate the production of curd-rich dairy products such as cheese. However, other studies have noted the bias towards the detection of dairy over other food-derived proteins in many instances [42], and we remain ignorant of the taphonomic impacts on food proteins in archaeological samples. Moreover, substantial challenges persist in the identification of proteins from ceramics (e.g. [68]), hence the need to focus on mineralized deposits and the limited number of samples used in this study, while the high survivability and extractability of absorbed lipids in the archaeological record has enabled the creation of a vast temporal and geographical corpus of lipid results. This study highlights the advantage of comparing protein results with those gained from lipid residue analysis and should provide a useful example to future studies. Further analyses using a larger dataset would be of benefit to understand both the method and the archaeological context. Moreover, additional experimental studies using larger sample sizes of modern vessels with known protein inputs will be instrumental in uncovering the biases associated with food protein entrapment, extraction and identification.

### 3.5. The significance of dairying multiple species

Previously, it has been proposed that the earliest dairy processing in the Near East was linked to cattle herding, based on correspondence between faunal and lipid data [28]. Here we show that this does not seem to be true for the Northern European Neolithic, nor previously at Çatalhöyük on the Anatolian plain [33], where, in both cases, dairy products from multiple taxa were processed, sometimes in the same vessel. Proteomic analysis of dental calculus also supports the exploitation of multiple taxa for dairy during the Neolithic, rather than a single species. Interestingly, evidence from Britain [13] and East Africa [38] indicates that this practice occurred as soon as domesticated animals were introduced. In Funnel Beaker contexts, the data are less forthcoming, however, Gron *et al*. [26] suggest that cattle were exploited for both meat and dairy through a complex multi-season birthing system, from the onset of the Neolithic, a practice probably brought about by incoming farmers into the region. Others too have also highlighted the close relationship between the Funnel Beaker culture, subsequent Globular Amphora culture and cattle [11], but this does not necessarily preclude dairying of other species. Cattle might have been revered more for their value ‘on the hoof’ as live animals rather than the products they produce, which were seemingly treated in the same way as those from other species. Indeed, some of the earliest evidence for wheeled transport in Europe derives from the TRB [69,70], including the depiction of a wagon on a TRB vessel from the Polish site of Bronocice [71]. Overall, we suggest that dairying at least initially encompassed a broader range of domesticated ruminants than previously thought, but further work is required to see if more specialized dairy economies developed later in prehistory.

## 4. Conclusion

In this study, evidence of dairy processing was identified at the TRB site of Sławęcinek in modern-day Poland, demonstrating the presence of Caprinae and Bovinae dairy products and therefore utilization of both of these taxonomies for their dairy products. The milk proteome identified in this study provided an opportunity to explore patterns of milk processing, beyond simply detecting the presence of dairy foods, and we note the dominance of caseins involved in curd-formation. This evidence suggests that TRB individuals may have been consuming dairy foods reduced in lactose, which has implications for our understanding of LP selection during this period. This study also yielded a valuable opportunity to compare protein and lipid-based approaches in organic residue analysis, where we significantly note that using Δ^13^C values less than or equal to −3.1‰ as a proxy for dairy fat may be underestimating the frequency of milk use in prehistoric pottery. We note that further experimental research is vital to identify key methodological biases between these two approaches in organic residue analysis.

## Data Availability

Proteomics data is available via the ProteomeXchange under accessions PXD037912 and PXD037913. Lipid data can be found in the Dryad Digital Repository: https://doi.org/10.5061/dryad.905qfttpt [72]. All other data can be found in this publication and its electronic supplementary material [73].
